# A protracted cholera outbreak among residents in an urban setting, Nairobi county, Kenya, 2015

**DOI:** 10.11604/pamj.2020.36.127.19786

**Published:** 2020-06-25

**Authors:** Hudson Taabukk Kigen, Waqo Boru, Zeinab Gura, George Githuka, Robert Mulembani, Jacob Rotich, Isack Abdi, Tura Galgalo, Jane Githuku, Mark Obonyo, Raphael Muli, Ian Njeru, Daniel Langat, Peter Nsubuga, Jackson Kioko, Sara Lowther

**Affiliations:** 1 Ministry of Health, Nairobi, Kenya,; 2 Field Epidemiology and Laboratory Training Program, Nairobi, Kenya,; 3 Ministry of Agriculture, Livestock and Fisheries, Nairobi, Kenya,; 4 African Field Epidemiology Network, Nairobi, Kenya,; 5 Department of Health, County Government of Nairobi, Nairobi, Kenya,; 6 Division of Disease Surveillance and Response, Ministry of Health, Nairobi, Kenya,; 7 Global Public Health Solutions, Atlanta, USA,; 8 US Centers for Disease Control and Prevention, Atlanta, Georgia, USA

**Keywords:** Cholera, outbreak, *Vibrio cholerae*, case-control, county, Nairobi, Kenya

## Abstract

**Introduction:**

in 2015, a cholera outbreak was confirmed in Nairobi county, Kenya, which we investigated to identify risk factors for infection and recommend control measures.

**Methods:**

we analyzed national cholera surveillance data to describe epidemiological patterns and carried out a case-control study to find reasons for the Nairobi county outbreak. Suspected cholera cases were Nairobi residents aged >2 years with acute watery diarrhea (>4 stools/≤12 hours) and illness onset 1-14 May 2015. Confirmed cases had Vibrio cholerae isolated from stool. Case-patients were frequency-matched to persons without diarrhea (1:2 by age group, residence), interviewed using standardized questionaires. Logistic regression identified factors associated with case status. Household water was analyzed for fecal coliforms and Escherichia coli.

**Results:**

during December 2014-June 2015, 4,218 cholera cases including 282 (6.7%) confirmed cases and 79 deaths (case-fatality rate [CFR] 1.9%) were reported from 14 of 47 Kenyan counties. Nairobi county reported 781 (19.0 %) cases (attack rate, 18/100,000 persons), including 607 (78%) hospitalisations, 20 deaths (CFR 2.6%) and 55 laboratory-confirmed cases (7.0%). Seven (70%) of 10 water samples from communal water points had coliforms; one had Escherichia coli. Factors associated with cholera in Nairobi were drinking untreated water (adjusted odds ratio [aOR] 6.5, 95% confidence interval [CI] 2.3-18.8), lacking health education (aOR 2.4, CI 1.1-7.9) and eating food outside home (aOR 2.4, 95% CI 1.2-5.7).

**Conclusion:**

we recommend safe water, health education, avoiding eating foods prepared outside home and improved sanitation in Nairobi county. Adherence to these practices could have prevented this protacted cholera outbreak.

## Introduction

Cholera is a rapidly dehydrating diarrheal disease caused by consuming water or food contaminated with a toxigenic serogroup of *Vibrio cholerae*. Cholera has a short incubation period of 12 hours to 5 days and the virulence of cholera can lead to rapid patient deterioration or trigger other explosive cholera outbreaks. Within hours of symptom onset, a previously healthy person can become severely dehydrated, electrolyte-depleted and develop shock. If not treated promptly, the patient may die within hours [1-3]. The control and prevention of cholera outbreaks require a robust and functional surveillance system for early detection, notification, correct disease burden estimation, reporting and response. In low and middle-income countries (LMICs), surveillance systems tend to be weak; outbreak detection is often delayed, which often results in large and sustained cholera outbreaks with high rates of attack, hospitalisation and fatalities [4,5]. Worldwide, there were nearly 1.4 to 4.3 million cases and over 100,000 deaths attributed to cholera in 2015 [1]. LMICs bear a bigger disease burden due to unplanned urbanisation, poverty, poor sanitation, population displacement, inadequate health structures and the lack of potable water [1,6,7]. In Africa, cholera has remained of a major public health concern since its re-emergence in 1970 [8]. From 1980 to 2011, sub-Saharan Africa reported 50% of the global cholera cases. Excluding the 2011 Haiti outbreak, sub-Saharan Africa accounted for 86% of the cholera burden and 99% of deaths worldwide [8-11].

Within sub-Saharan Africa, Kenya has experienced frequent cholera outbreaks, with more than 15 discrete outbreaks from 1971 to 2010. Populations living in Nairobi´s urban informal settlements were particularly affected by cholera outbreaks occurring during 2005 and 2010 [12-14]. Nairobi county, which has over 60% of its population residing in >180 informal settlements, last reported a cholera outbreak in 2009-2010 [15]. On the 6^th^ of January 2015, the Kenya Ministry of Health (MoH) received a notification from Nairobi´s Kamukunji sub-county disease surveillance office of a suspected cholera case following an admission of a 40-year-old man at Kenyatta National Referral Hospital. Located in Nairobi county, Kenyatta National Referral Hospital is a public referral and teaching hospital with 1,800 beds. The case-patient presented with severe dehydration and shock following acute diarrhea and vomiting. Stool specimens collected and tested at the Kenya National Public Health reference laboratory on the 7^th^ of January 2015 confirmed toxigenic *Vibrio cholera* O1 subtype Inaba.

Further review of surveillance data and an active case search by residents from the Kenya Field Epidemiology and Laboratory Training Program (FELTP) revealed four cholera cases in Nairobi county during December 2014. All four cases met the MoH´s case definition of suspected cholera: severe dehydration or death from acute watery diarrhea with >4 episodes of loose stool in 12 hours in a patient aged ≥5 years, or an acute watery diarrhea in a patient aged ≥2 years in an area where there is a cholera outbreak. The earliest case identified was a 34-year-old female from Starehe sub-county in Nairobi county who had onset of symptoms on the 26^th^ of December 2014. She was admitted for 2 days at a private facility, managed and discharged. During 2015, Nairobi county continued reporting an increasing number of cholera cases and these spread into previously unaffected sub-counties. Because of this protracted reporting, residents from the FELTP investigated the outbreak with the objectives of determining outbreak magnitude, characterising the cholera cases and identifying risk factors associated with the outbreak in Nairobi county, Kenya, 2015.

## Methods

**Figure 1:**
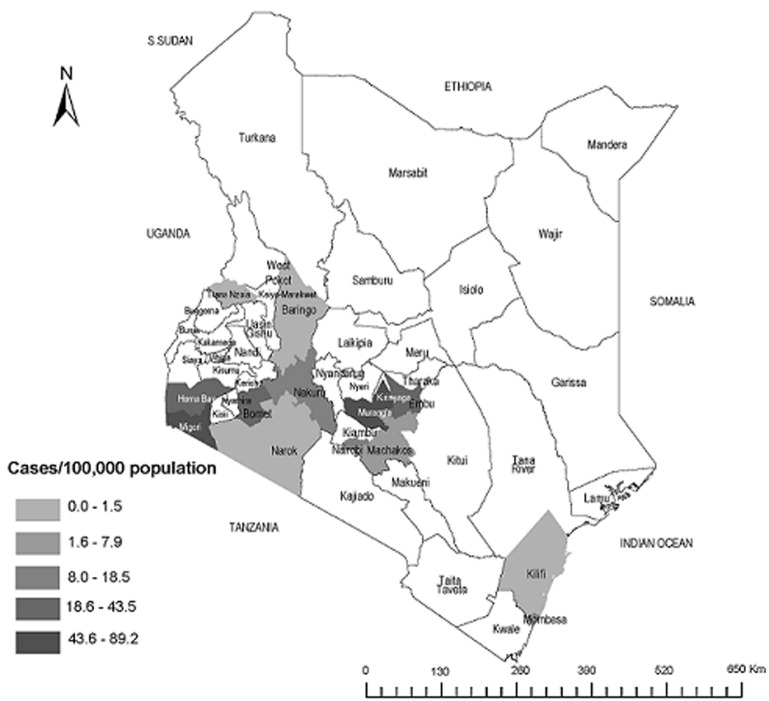
spatial distribution of cholera cases per 100,000 population reported in 14 of the 47 counties in Kenya, December 2014 - June, 2015

**Figure 2:**
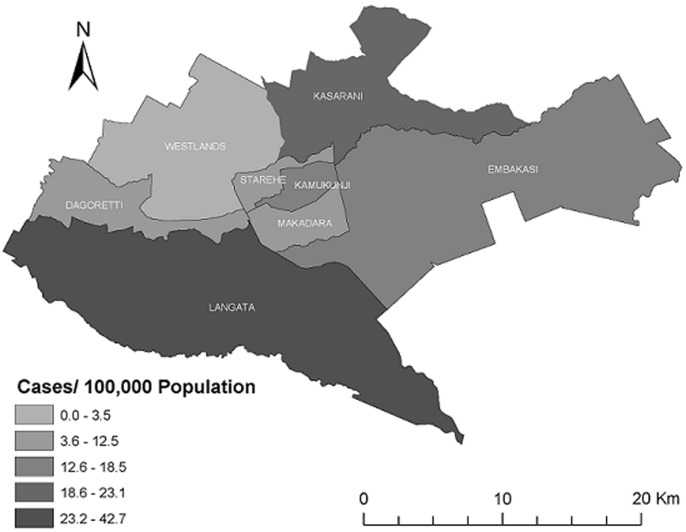
spatial distribution of cholera cases per 100,000 population reported per sub-county, Nairobi county, Kenya, December, 2014 - June, 2015

**Investigation setting:** Nairobi county the smallest (696km^2^area) and most populous of the 47 counties of Kenya (4,232,087 persons, Kenya National Bureau of Statistics, 2015), has eight administrative sub-counties (Figure 1, Figure 2). Water and sewerage services are managed by the Nairobi county water and sewerage company, a government entity. Nairobi is the capital and largest city of Kenya with ≈60% living in informal settlements. The United Nations defines an informal settlement as a contiguous settlement where residents have inadequate quality of or a lack of the following: housing, access to safe water, sanitation, secure tenure, durability of housing and living area. In addition to having limited water supply, water in informal settlements may come from unregulated sources such as street vendors, most at times not adhering with water quality testing procedures, which could compromise water quantity and quality.

**Hypothesis generation:** during the initial investigation, we performed a descriptive analysis of MoH national and county cholera surveillance data and conducted informal in-person interviews with the county healthcare managers, healthcare workers, case-patients and the public. County officials were concerned that the majority of the cholera case-patients resided in informal settlements where sanitation is poor and water supply inadequate and unregulated. We observed that hospitals were congested, deficient in quantities of required personal protective equipment and designated beds for cholera patients and had relatives freely interacting with suspected cholera patients. We noted that the water supply in the informal settlements was erratic, due to frequent rationing. We observed congested housing, open disposal of waste, poorly-connected leaky water pipes, discharging sewage lines, clogged drainage systems and food vending. Given these observations, we generated our research questions to be tested using a case-control study. We hypothesised that environmental, sanitation and hygiene practices were contributing to the cholera cases in Nairobi informal settlements.

**Environmental sampling:** during the outbreak investigation, we collected water samples from Nairobi county communal water points (fixed water points serving several households), we selected based on where the majority of the case-patients stated that they fetched water for daily household use. Ten water samples from 10 different communal water points were collected. We used sterile bottles provided by Kenyan National Public Health Laboratory Services (NPHLS). We transported samples using cooler boxes within 3 hours for water microbiological and quality analysis at NPHLS.

**Laboratory sampling:** stool and rectal swab specimens from case-patients were collected by healthcare workers at the cholera treatment centres. Specimens were transported using Cary-Blair media to NPHLS for laboratory analysis.

**Study design:** we conducted a descriptive analysis of the cholera outbreak line list from the MoH and Nairobi county databases and followed with a frequency-matched case-control study in Nairobi county. We consolidated data from eight sub-counties in Nairobi county with the overall Nairobi county cholera line list during December 26^th^, 2014 to June 8^th^, 2015. We compared this enhanced, overall Nairobi county line-list with the nationally-reported disease surveillance and response unit (DSRU) cholera database to remove duplicates and correct any other data errors with regard to case demographics and clinical information. The final line list included variables on name, age, sex, residence, date of diarrhea onset, date of admission, case management, laboratory diagnosis and patient clinical outcomes.

**Case-control study:** we conducted a matched case-control study. We frequency matched by age group and sub-county of residence of confirmed and probable cases with two controls to identify risk factors associated with being a cholera case in Nairobi county. Age groups were 2-4, 5-15, 16-30, 31-45 and >45 years.

**Case definition:** a suspected case-patient was a Nairobi county resident living in the selected sub-counties, aged ≥2 years old with acute onset of watery diarrhea of ≥4 loose stools in a 12 hour period, with or without vomiting during the 1^st^-11^th^ of May 2015. A probable case occurred in any patient that had an epidemiological linkage to a confirmed case. Confirmed cases were those with laboratory isolation of *Vibrio cholerae* from stool culture that met either the probable or suspected case definition. Controls were selected as being in the same age group and having lived in same selected sub-counties as cases, with no history of watery diarrhea of ≥4 loose stool in 12 hours or vomiting since the onset of the outbreak, on the 20^th^ of December 2014. Severely sick patients who were not able to answer to interview questions were excluded from the study.

**Sample size calculation:** we assumed a two-tailed confidence level at 95%, 80% power, a desired odds ratio of 3.0 between cases and controls and a 49% prevalence of exposure for hand washing before eating among controls [16,17]. We calculated a minimum sample size of 156 using Fleiss´ formula in Epi-Info 7^TM.^(US Centers for Disease Control and Prevention [CDC]-Atlanta).

**Selection of cases and controls:** from the Nairobi county line list, four of the eight sub-counties were selected according to the proportion of the cholera case burden. From the four sub-county line list, we selected cases by systematic random sampling. Cases in the hospital wards were administered a structured questionnaire via an in-person interview. We used telephone contacts to trace case-patients who had been discharged from the health facility. Sub-county disease surveillance officers, community health workers (CHWs) and community health volunteer heads assisted the team in locating households. At each household, we recorded water, hygiene, environmental and sanitation characteristics. For each case-patient interviewed, we identified the two control subjects by spinning a bottle near the selected case-patient´s house and selected the immediate household to the direction of the bottle. The second control was selected by spinning the same bottle at the door step of the first control and the immediate household on the bottle´s direction was selected. Where there was more than one eligible control of same age category in a household, we numbered the potential controls and randomly selected one. If an eligible study participant was not present at the time of the study, we made three visits to the household within 24 hours before replacement. In cases where selected households were without eligible controls, we excluded and the bottle spinning was repeated. We interviewed two controls on the same day as their case.

**Data collection:** we collected data on occupation, age, sex, residence, religion and factors related to exposure and/or cholera transmission such as: hand washing practices, water sources, water treatment and storage, food, hygiene and sanitation practices for both cases and controls. We recorded clinical information for cases from their hospital records.

**Data management:** we managed and analysed data using Epi-Info 7^TM.^(CDC-Atlanta) and Microsoft Excel 2013 (Microsoft Office, Seattle, USA). We calculated descriptive statistics for the characteristics of cases from cholera surveillance data. Overall sex and sub-county cholera attack rates (AR) were obtained by dividing the number of cases with baseline national, county and sub county population estimates, (Kenya National Bureau of Statistics [KNBS], 2015). For analysis of sub-county specific attack rates, we used 2013 sub-county boundaries: Langata (Langata and Kibra), Dagoretti (Dagoretti South and North), Embakasi (Embakasi North, South, Central, West and East), Kasarani (Kasarani, Ruaraka and Mathare), Starehe, Kamukunji, Westlands and Makadara. Case-fatality rates (CFR) were calculated by dividing the number of deaths by total numbers of suspected cases. The proportions of water samples with coliforms and *E. coli* was also reported.

**Case-control study:** we performed univariate, bivariate and stratified analyses. We performed chi-square tests to identify factors associated with the outbreak and calculated odds ratios (OR) and 95% confidence intervals (CI). We included factors with p<0.2 in a logistic regression model. We used stepwise forward selection method and variables with a p<0.05 were considered to be independently associated with being a cholera case-patient.

**Ethical consideration:** consent was required before questionnaire administration. Study objectives and intended use of the results in prevention and control of diarrheal diseases were explained to study participants. The investigation was considered a response to a disease of public health by the Ministry of Health; therefore, the investigation did not require institutional review board approval. The investigation protocol was approved by the MoH. Permission was granted from DSRU and the county health managers. The investigation was determined to be non-research by the Centers for Disease Control and Prevention (CDC) Center for Global Health. We de-identified any identifying information before reporting.

## Results

**Figure 3:**
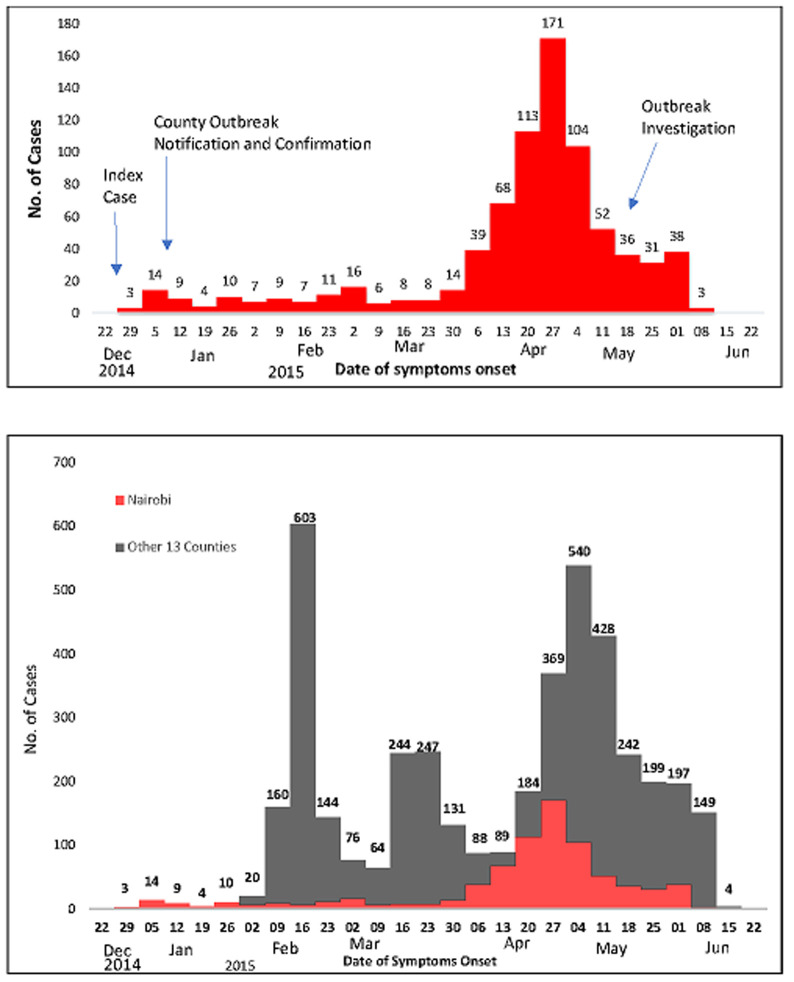
cholera epidemic curve of Nairobi county in comparison with the national (14 counties) cholera outbreak (N =4218) by date of symptoms onset, December 2014 - June 2015, Kenya, 2015

From December 2014 through June 2015, 4,218 cholera cases were reported including 282 (6.7%) confirmed cases and 79 deaths (case fatality rate [CFR] = 1.9%, ranging from zero in Narok county to 5.3% in Mombasa county) from 14 (30%) of Kenya´s 47 counties (Figure 1). Of the 4,218 cases, 2,315 cases (56%) were reported in three counties: Migori (22%), Nairobi (19%) and Muranga (15%) (Table 1). The national epidemic curve had multiple peaks of varying magnitudes as cholera cases spread between counties, with the largest peak occurring in February, 2015. Beginning June 2015, the outbreak in three counties (Homa Bay, Migori and Bomet) had died out, defined as reporting zero cases 14 days after the last reported case (Figure 3). During the same period (December 2014 through June 2015), Nairobi county reported 781 cases, representing 19% of all the cholera cases reported in Kenya. Cholera attack rate was 18 cases per 100,000 persons (781 cases/4,232,087 persons) with 20 deaths (CFR = 2.6%). Of the 781 cases, there were 607 (78%) hospitalisations. A total of 55 cases (7.0%, [53 Ogawa, 2 Inaba]) were laboratory-confirmed (Table 1). Nairobi county CFR was higher than the national CFR of 1.9% and the WHO accepted rate of ≤1%. Incidence was higher among persons aged 15-24 years (24 cases/100,000 persons) than other age categories. Overall, 473 (61%) were male, with the highest incidence among males being in those aged 15-34 years; among females, the highest incidence was among those aged 35-44 years (Table 2).

**Table 1:** number (N=4218) and percentage of reported cholera cases, number confirmed, number of deaths, and case-fatality rate - 14 counties, Kenya, December 26, 2014-June 10, 2015

County	Reported cases (%)	Attack rate (case/100,000 persons)	Percent of cases laboratory confirmed	Case Fatality Rate
Migori	915 (22)	89.0	1.6	1.2
Nairobi	781 (19)	19.0	7.0	2.6
Muranga	619 (15)	59.0	4.7	0.8
Homa Bay	469 (11)	44.0	8.5	1.1
Kirinyaga	378 (9)	65.0	0.3	0.5
Bomet	272 (6)	32.0	15.1	0.7
Nakuru	266 (6)	14.0	13.5	3.8
Mombasa	189 (5)	17.0	13.2	5.3
Embu	179 (4)	33.0	2.8	1.7
Machakos	59 (1)	5.0	13.5	5.1
Baringo	52 (1)	8.0	21.1	1.9
Kilifi	21 (1)	2.0	47.6	4.8
Narok	13 (0.3)	1.0	30.0	0
Trans Nzoia	05 (0.2)	1.0	60.0	0
Total	4218 (100)	24.0	6.7	1.9

**Table 2:** cholera attack rates (AR) stratified by sex and age, Nairobi county, Kenya 2015

Age group	Total cases	Total population (1000)	Overall attack rate	Male cases	Male population (1000)	Male attack rate	Female cases	Female population (1000)	Female attack rate
0-4	107	630	17.0	47	316	15.0	51	314	16.0
5-14	123	793	16.0	65	388	17.0	53	405	13.0
15-24	185	774	24.0	111	336	33.0	77	438	18.0
25-34	184	1061	17.0	129	499	26.0	56	562	10.0
35-44	113	560	20.0	74	314	24.0	49	246	20.0
>45	69	413	17.0	47	241	20.0	22	173	13.0
Overall	781	4232	19.0	473	2094	23.0	308	2138	14.0

Population data source-Kenya National Bureau of Statistics (KNBS), 2015

**Figure 4:**
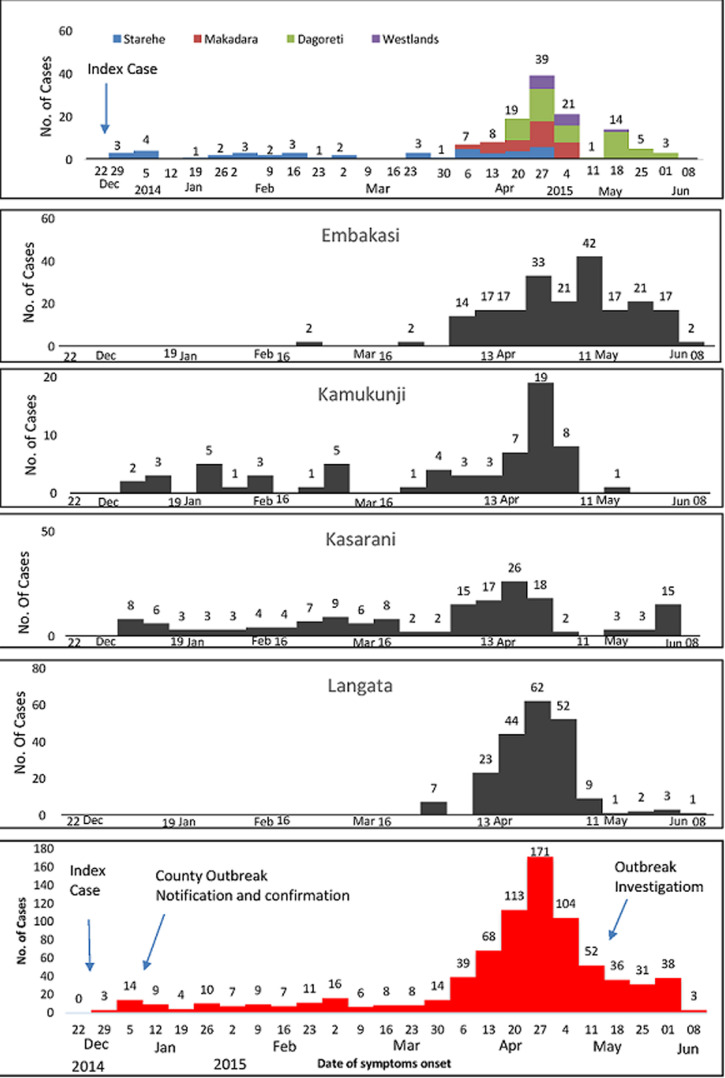
an integrated Nairobi county cholera epidemic curve (N=781) and sub county panels by date of symptom onset showing persistent case reporting and multiple peaks, Nairobi county, Kenya, 2015

Illness onset for the index case-patient was on the 26^th^ of December 2014 and cases continued to be reported until June 2015. There was a delay of 12 days from the onset of symptoms of the index case to the initial outbreak detection, notification and laboratory confirmation at the county level (Figure 3). The urban Nairobi epidemic curve was protracted, showing multiple small peaks of varying sizes during January to March. Cholera spread between sub-counties with the largest and most explosive peak occurring in April, which coincided with when Dagoreti, Makadara, Westlands, Langata and Embakasi sub-counties all had cases being reported (Figure 3). Langata, Kasarani and Kamukunji sub-counties had above-average cholera incidence and Langata sub-county had an attack rate that was more than double that of the overall Nairobi county attack rate (18.5 cases/100,000 population) (Table 3, Figure 4). Within these affected sub-counties, all of the cases occurred in informal urban settlements. Of the 10 water samples collected at the Nairobi county communal water points in May 2015, 2 (20%) were satisfactory for human consumption (WHO class I and II) and 7 (70 %) had 5 to >180 coliforms/100mls of water (class III and IV) and were unsatisfactory for human consumption. One water sample had *E. coli* levels >40/100mls of water.

**Table 3:** cholera attack rate (AR) per sub-county, Nairobi county, Kenya, 2015

Sub-County	Population (1000)	Cases (%)	Attack rate (cases/100,000 persons)
Langata	478	204 (26.0)	43.0
Kasarani	709	164 (21.0)	23.0
Kamukunji	354	66 (9.0)	19.0
Embakasi	1248	205 (26.0)	16.0
Dagoretti	445	55 (7.0)	12.0
Starehe	371	44 (6.0)	12.0
Makadara	295	31 (4.0)	11.0
Westlands	332	12 (2.0)	4.0
Total	4232	781 (100)	18.5

Population source: Kenya National Bureau of Statistics 2015

**Case-control study:** we enrolled 52 cases and 104 controls. Of the enrolled study participants, 89% of the cases had less than 12 years of education. Among enrolled case-patients, the median duration of symptoms before seeking treatment at the hospital was 1 day (range 1-10 days) while median hospital stay was 2 days (range 1-9 days). Of the 52 cases selected for interviews, 5 (9.6%) reported having been informed of their laboratory result at the hospital (Table 4). In bivariate analysis, self-reported exposures associated with cholera cases included: eating food prepared outside of the home, close contact with a person who had watery diarrhea within five days, eating cold leftover foods in the last five days, a lack of health education on diarrheal illness or cholera in the last 6 months, non-treatment of drinking water, not washing hands before eating or after visiting toilets and fecal contamination observed within the household compound by the survey team. We found that those who reported washing fruits were protected against infection (Table 5). A stratified analysis did not identify evidence of confounding of associated variables with level of education (data not shown). Communal water taps were the main source of drinking water for 98 (94%) of the participants; this did not differ between cases and controls.

**Table 4:** characteristics of cholera cases and controls, Nairobi county, Kenya, 2015

Variable	Characteristic	Cases n=52 (%)	Controls n=104 (%)	P-value
Sex	Male	29 (56.0)	57 (55.0)	Reference
	Female	23 (44.0)	47 (45.0)	0.91
Age (years)	2-4	06 (12.0)	13 (13.0)	0.51
	5-15	09 (17.0)	17 (16.0)	0.61
	16-30	14 (27.0)	29 (28.0)	0.48
	31-45	17 (33.0)	37 (36.0)	0.42
	>45	06 (12.0)	08 (08.0)	Reference
Sub-County	Langata	35 (67.0)	70 (67.0)	0.65
	Embakasi	12 (23.0)	24 (23.0)	0.63
	Makadara	03 (06.0)	06 (06.0)	0.57
	Kamukunji	02 (04.0)	04 (04.0)	Reference
Occupation	Formal employment	21 (40.0)	54 (52.0)	Reference
	Informal employment	31 (60.0)	50 (48.0)	0.18
Religion	Christian	45 (87.0)	88 (85.0)	Reference
	Muslim	07 (13.0)	16 (16.0)	0.75
Education	≥12 years education	46 (89.0)	71 (68.0)	Reference
	>12 years education	06 (12.0)	33 (32.0)	0.006

**Table 5:** risk factors associated with being a cholera case, Nairobi county, Kenya, 2015

Risk Factors	Case n=52 (%)	Controls n=104 (%)	OR	95% CI	Final aOR	model 95% CI
Less than 12 years of schooling (secondary)	46 (89)	71 (69)	3.6	1.4-9.2	--	--
Not re-heating left-over food	13 (33)	06 (08)	5.7	1.9-16.5	--	--
Not treating drinking water last 5 days	45 (87)	41 (39)	9.9	4.1-24.0	6.5	2.3-18.8
Reported eating outside of the home	34 (65)	40 (39)	3.0	1.5-6.1	2.4	1.2-5.7
Washing fruits	17 (33)	78 (75)	0.1	0.1-0.3	--	--
Not washing hands with soap/water before meals	27 (55)	26 (25)	3.5	1.7-7.3	--	--
Not washing hands with soap/water after defecation	22 (45)	28 (27)	2.2	1.1-4.5	--	--
Observed signs of open defecation within compound	46 (89)	58 (56)	6.1	2.4-15.5	--	--
Contact with persons with diarrhea last 5 days	14 (27)	11 (11)	3.1	1.3-7.4	--	--
A lack of health education on diarrheal illness or on cholera last 6 months	42 (82)	41 (41)	6.7	2.9-15.2	2.4	1.1-7.9

n- Number of cases or controls, % - Percent, OR - Odds Ratio, CI - Confidence interval, aOR **-** Adjusted odd ratio

From the logistic regression model, we identified that independent risk factors associated with being a cholera case were: drinking untreated communal water, a lack of health education on diarrheal illnesses and cholera in the last 6 months and eating food that was prepared outside of their home in the five days before illness onset (Table 5). In response to our investigations within Nairobi county, we disseminated our findings to the team leads at the health facilities, sub-counties, county and the MoH managers. A multi-pronged water, sanitation and hygiene (WASH) team consisting of staff from the National and county level and other stakeholders was activated. The advocacy, communications and social mobilization team from the MoH promotion unit designed health promotional messages in print and electronic formats targetting the affected communities. Community health extension workers (CHEWs) and volunteers distributed chlorine commodities to treat communal water at households and the Nairobi water and sanitation company supplied safer water. Routine surveillance of water sources and water quality analysis on the levels of residual chlorine of the communal water sources was to done weekly. Also, the public health department through health education and licensing put strategies to regulate street food vending.

## Discussion

This national cholera outbreak grew in magnitude as it spread to involve several newer counties during the reporting period and had a higher CFR than the WHO standard, with a CFR that varied between counties. Likely contributors to the urban Nairobi county protracted cholera outbreak were not treating ones household drinking water, inadequate personal and environmental hygiene, poor sanitation, eating contaminated food outside the home and a lack of health education. The majority of the cases were caused by *Vibrio cholerae* O1 subtype Ogawa, biotype El Tor, the presence of both Ogawa and few Inaba subtypes suggests different sources of introduction and subsequent circulation [12,18,19]. This is in contrast to outbreaks during 2005 and 2009-2010 during which the predominant subtype was Inaba [13,20]. The CFR was lower nationally and in Nairobi county during this 2015-2016 outbreak compared to the 2009-2010 cholera outbreak in the Western Kenya region and East Pokot county [21]. This lower CFR was likely because of the collaboration of health sectors and stakeholders in implementing treatment, control and prevention interventions. Treatment centres were set up in cholera epicentres, potable water and water treatment commodities like aqua tabs were provided, handwashing equipment and soap was made available at the community level, a comprehensive cholera risk communication strategy was initiated and extra health staff were deployed to the community hospitals.

Despite these interventions the CFR was still above the WHO recommended 1%, which assumes appropriate and timely case management with rehydration, with administration of oral rehydration solutions (ORS). The higher than recommended CFR indicates delays in health care access, limitation in timely detection or insufficient health services typical of informal settlements in LMIC. The high hospitalization rate, might suggest even not severe cases were admitted and reported. As part of the outbreak response, health promotion officers from the national MoH, the Nairobi county department and the community social mobilizers conducted health promotion activities in Nairobi county. A survey which enrolled a total of 1,418 randomly-selected households in the affected sub-counties, found increased awareness on cholera and knowledge of where and when community members should seek health care (Kenya FELTP, unpublished data). A study done by K-FELTP showed, despite community awareness on cholera, access to safe water was inadequate with over half of stored and source water samples inadequately treated [22]. The health promotion activities and the increased awareness among communities in Nairobi county could explain the high hospitalization rate during the later phase of the outbreak. The high hospitalization could also indicate that hospital staffs did not adequately triage and use oral rehydration solution (ORS) to manage the majority of cholera patients who could have only needed ORS as per the Ministry of Health or WHO cholera treatment guidelines [22].

High attack rates might relate to high population density among residents who shared utilities in informal settlements, or the cholera strains during this outbreak could have been more virulent [12,23]. The rapid spread of this 2014-2016 outbreak throughout all sub-counties of Nairobi might indicate weak surveillance and a failure by the health system to detect and respond. Delayed detection and small sub-county outbreak peaks from January to March allowed cholera to become entrenched in communties and explode in neighboring sub-counties, in early April when a larger peak involving Langata and Embakasi sub-counties appeared. This differs from the 2010 cholera outbreak, when the few Nairobi urban settlements reported confined cholera cases were contained in a timely fashion [13,24]. Persons living in Nairobi informal settlements were likely not to treat one’s household drinking water, a key driver for this outbreak. Untreated drinking water as a transmission route for cholera is a major challenge for residents of informal settlements where water is scarce or frequently get interrupted and water sources are are of low quality and unregulated, insufficient quantity. During this outbreak, we observed poor integrity of the water piping materials serving the communal water points, the pipes were running in close proximity to sewage lines, there was leakage in pipes, feacal disposal was generally inadequate in most households, the toilet facilities were fewer. Due to the intermittent and unregulated water supplies, water quality testing was may not have been routinely done.

The combination of these factors might have contributed to contamination of water sources at shared water reservoirs. Treating drinking water is a cheap and effective intervention in reducing waterborne illnesses [17], which was not consistently done by residents in the outbreak-affected communities. Nairobi county cholera cases were more likely to have reported eating food that was prepared outside the home compared to non-ill controls. Ill persons might tend to blame their illness on food prepared outside the home and our investigation did not trace back individual food vendors to confirm these vendors as a source of illness. The reported consumption of food outside the households might indicate unsafe food practices and the several unregulated streetside food-vending within the informal settlements could have lead to illness among cases. Streetside food vendors and their customers may not adhere and maintain critical food safety control points as they relate to food preparation and handling in streetside environments [15]. The study team observed that food vendors remained open during this outbreak despite county health management efforts to control street vending. Regulating street food vending is a challenge to policy makers, since street vendors are diverse, mobile and serve large populations. Challenges in regulating food vending in informal settlements has been documented during other cholera outbreaks in South Sudan, Uganda and Haiti [25-28]. Nairobi county cases were more likely than controls to remark on a lack of health education on diarrheal illness.

Persons who lack knowledge about cholera may not be able to apply promotive, preventive and health control measures and thus remain at greater risk of contracting cholera. Previous studies conducted in Kenya, Zimbabwe and Haiti also identified knowledge gaps as drivers of cholera outbreaks [22,23,28-30]. Following our investigation, a study done found that CHEWs played a critical role in providing health education about cholera prevention and control [31]. Our study had some limitations. Recall bias is possible such that ill cases might have differing recall of their exposures than controls. We attempted to limit recall bias by restricting questions on exposures within 5 days before symptom onset. Misclassification of outcome status is possible, since control selection was based on clinical symptoms and some controls might have been asymptomatically infected or mild gastrointestinal symptoms, knowing over 75% of persons who ingest toxigenic *V. cholerrae* O1 typically have no diarrhea. To limit this by requesting stool specimens from both cases and asymptomatic controls for serological IgM or IgG testing was not feasible in this resource-poor outbreak setting. Lastly, we matched study participants by place of residence, which limited our analysis of place as a potential risk factor.We did not differentiate a first case or subsequent household cases, hence we could not be able to suggest whether the exposure (contamination) was at community or household level. However, recruitment from different settlements and heterogeneity of our study participants provide valuable insights that allow an understanding of risk factors of cholera outbreaks in informal settlements.

## Conclusion

Informal settlements in the sub-counties frequently experienced cholera outbreaks sustain transmission. Drinking untreated water, inadequate personal and environmental hygiene, poor sanitation, eating food outside homes and a lack of health education on risks have likely contributed to this prolonged outbreak. Provision of safe water, improvement in sanitation, health education and adherence to appropriate food handling are needed, which county health officials and national officials can address under the regulation of the Kenya public health act.

### What is known about this topic

- Populations residing in informal settlements with inadequate water and sanitation remain at a higher risk of cholera epidemics;
- Cholera is very virulent, easily transmitted by consuming contaminated food and/or water, cholera can affect a large area in a short time period if control measures are not put in place.

### What this study adds

- The Nairobi county cholera outbreak had delays in detection and confirmation. The outbreak continued over 6 months, spreading to all the sub-counties. This could suggest implementation of the outbreak surveillance system remains a challenge to most counties in Kenya;
- Untreated water sources continue to remain a risk factor in informal urban settings. This was a strong risk factor during the Nairobi cholera outbreak.
